# Total Glucosides of Paeony Alleviate Cell Apoptosis and Inflammation by Targeting the Long Noncoding RNA XIST/MicroRNA-124-3p/ITGB1 Axis in Renal Ischemia/Reperfusion Injury

**DOI:** 10.1155/2020/8869511

**Published:** 2020-11-24

**Authors:** Fang Chen, Yi Hu, Yuetao Xie, Zonghui Zhao, Lin Ma, Zhili Li, Wanlong Tan

**Affiliations:** ^1^Department of Urology, Nanfang Hospital, Southern Medical University, Guangzhou, Guangdong 510515, China; ^2^Department of Anesthesiology, Shenzhen Children's Hospital, Shenzhen, Guangdong 518038, China

## Abstract

**Objective:**

Renal ischemia/reperfusion injury (RI/RI) is the main cause of acute kidney injury. Total glucosides of paeony (TGP) are a traditional Chinese medicine. This study was aimed at exploring the role of TGP in RI/RI and its underlying mechanism of action.

**Methods:**

Rat RI/RI models were constructed by surgical operation. Serum creatinine (Scr) and blood urea nitrogen (BUN) were used to evaluate renal function. The levels of proinflammatory cytokines were detected by ELISA. RI/RI was simulated by hypoxia/reoxygenation (H/R) treatment in renal cells *in vitro*. The lncRNA XIST (XIST) expression was analyzed by qRT-PCR. Then, the viability and apoptosis of renal cells were detected by MTT and flow cytometry assay. Additionally, dual-luciferase reporter assay was used to determine the interactions among XIST, microRNA-124-3p (miR-124-3p), and ITGB1.

**Results:**

TGP improved renal function and inhibited inflammatory responses after RI/RI. XIST expression was highly expressed in rat RI/RI models and H/R-treated renal cells, whereas treatment with TGP downregulated the XIST expression. Additionally, TGP increased viability and attenuated apoptosis and inflammation of H/R-treated renal cells via inhibiting XIST. Moreover, XIST was competitively bound to miR-124-3p, and ITGB1 was a target of miR-124-3p. miR-124-3p overexpression or ITGB1 inhibition rescued the reduction effect on viability and mitigated the promoting effects on cell apoptosis and inflammation caused by XIST overexpression in H/R-treated renal cells.

**Conclusions:**

*In vivo*, TGP attenuated renal dysfunction and inflammation in RI/RI rats. *In vitro*, TGP inhibited XIST expression to modulate the miR-124-3p/ITGB1 axis, alleviating the apoptosis and inflammation of H/R-treated renal cells.

## 1. Introduction

Renal ischemia/reperfusion injury (RI/RI) is a dynamic process including inflammation and some regulators in a complex interaction [1]. It often occurs during diverse clinical and surgical settings, which is a main cause of acute kidney injury [2]. RI/RI induces multiple degrees of injury to renal tissues, and the morbidity and mortality remain high [3, 4]. Thus, development of an effective strategy to alleviate RI/RI is imperative. Previous studies have shown that traditional Chinese medicines (TCMs) such as berberine nanoparticles [5], honokiol [6], and polydatin [7] can be effective treatments in RI/RI. Total glucosides of paeony (TGP), a TCM extracted from the dried roots of P. lactiflora Pall, mainly contain albiflorin, paeoniflorin, benzoyloxypaeoniflorin, hydroxy paeoniflorin, and other monoterpene glycosides [8]. TGP has been used to alleviate renal injury in kidney diseases. TGP alleviates early kidney damage by reducing levels of proinflammatory cytokines in the renal function of diabetic rats [9]. TGP improves renal function and attenuates the inflammatory responses in diabetic nephropathy [10]. Additionally, TGP treatment protects against ischemia/reperfusion injury (IRI) in different organs, such as in cerebral [11] and cardiac organs [12]. However, the underlying mechanism of TGP in the regulation of RI/RI is still unknown.

Recently, numerous studies have indicated that long noncoding RNAs (lncRNAs) are tightly related to IRI, such as lncRNA MALAT1 in myocardial IRI [13], lncRNA AK139328 in hepatic IRI [14], and lncRNA np_5318 in RI/RI [15]. It has been well documented that lncRNA X-inactive-specific transcript (XIST) takes part in the regulation of many kidney diseases. For instance, XIST knockdown ameliorates the renal interstitial fibrosis via enhancing miR-93-5p in diabetic nephropathy [16]. Silencing of XIST ameliorates acute kidney injury by decreasing miR-142-5p and enhancing PDCD4 expression [17]. XIST expression is highly expressed in membranous nephropathy, and XIST silencing attenuates podocyte apoptosis [18]. Despite these reports, the role of XIST in RI/RI remains to be elucidated.

Recent evidences have suggested that microRNAs (miRNAs) participate in the pathogenesis of RI/RI. miR-21 affords a kidney-protective effect against RI/RI through decreasing renal cell apoptosis [19]. miR-26a alleviates renal inflammatory response and ameliorates renal function in RI/RI [20]. As a regulative gene, miR-124 was proven to participate in the development of IRI. For example, miR-124 obviously reduces the H_2_O_2_-induced apoptosis of hepatic cells to improve hepatic recovery in hepatic IRI [21]. miR-124 derived from M2-EXO suppresses neuronal apoptosis and neural deficits, protecting the mouse brain against IRI [22]. Interestingly, miR-124-3p overexpression rescues the inflammatory response in myocardial IRI and apoptosis in cardiac myocyte caused by lncRNA ROR overexpression [23]. Nonetheless, the regulatory relationship between XIST and miR-124-3p in RI/RI remains undefined.

Existing research has demonstrated that diverse integrins play a pivotal role in kidney disease [24]. Integrins are a family of transmembrane receptors, and the integrin beta 1 (ITGB1) is a subunit of integrins [25]. ITGB1 is a critical factor in the regulation of renal structure and function [26, 27]. tPA accelerates the LRP-1-mediated recruitment of ITGB1, promoting the development of renal fibrosis [28]. Alpha 8 integrin, one kind of ITGB1, affects renal development and the susceptibility to kidney damage in mice [29]. However, the relationship between XIST and ITGB1 in RI/RI is still unclear.

In this study, we constructed rat RI/RI models and hypoxia/reoxygenation- (H/R-) treated renal cell models. Then, we evaluated the role of TGP in RI/RI rats. Additionally, we investigated whether TGP controlled the cell apoptosis and inflammation via modulating XIST/miR-124-3p/ITGB1 axis in RI/RI. Our study offers insight into mechanisms underlying modulations of TGP and XIST and provides a novel therapeutic target for RI/RI.

## 2. Materials and Methods

### 2.1. Animals

Male Sprague-Dawley (SD) rats (8 weeks old) were purchased from Beijing Vital River Laboratory Animal Technology Co., Ltd. The rats were fed standard chow and water and maintained under temperature-controlled conditions with an artificial 12 h light/dark cycle. This study was performed with the approval of our hospital ethics committee.

### 2.2. RI/RI Rat Model Establishment

After one week of adjustment, the RI/RI rat model was established. Simply, SD rats were anesthetized with intraperitoneal injection of pentobarbital sodium (50 mg/kg). A small incision was made through the medioventral line and exposed the right renal system. The right renal system was liberated, and nephrectomy was implemented. The left renal system was exposed, and the kidney artery was ligated with a silk suture. The kidney received the reperfusion after 45 min ischemia, and the wounds were closed using a medical suture. Rats in the sham group (*n* = 10) were given all the procedures except ligation. Then, the RI/RI rats were divided into three groups (*n* = 10): RI/RI rats without treatment served as the I/R group, RI/RI rats that received intragastrical administration of 0.9% sterile NaCl every 24 h for 7 days before ischemia acted as the negative control (NC) group, and RI/RI rats that received intragastrical administration of 200 mg/kg TGP (suspended in 0.9% sterile NaCl) every 24 h for 7 days before ischemia were regarded as the TGP group.

### 2.3. Sample Collection

At eight weeks, the rats were fasted 12 h and subsequently anesthetized by intraperitoneal injection of sodium pentobarbital (50 mg/kg) and sacrificed by cardiac puncture. Blood and kidney samples were collected for subsequent experiments. The blood samples were allowed to stand at 25°C to coagulate, and then the serum was obtained by centrifugation at 3000 rpm for 10 min at 4°C. The serum was stored at -80°C refrigerator. The renal tissues were removed and put into liquid nitrogen.

### 2.4. Evaluation of Renal Function

Levels of serum creatinine (Scr) and blood urea nitrogen (BUN) were measured one day following renal ischemia by an automatic biochemical analyzer (Hitachi, Tokyo, Japan).

### 2.5. Haematoxylin-Eosin (HE) Staining

Renal tissues were fixed in 4% paraformaldehyde for 24 h, embedded in paraffin, cut into 4 *μ*m thick sections, dewaxed in xylene, and rehydrated with ethanol. Sections were then stained with haematoxylin for 2 min and with eosin for 2 min. Using light microscopy, the histological injury was observed.

### 2.6. Cell Culture and H/R Treatment

The rat renal tubular epithelial cell line NRK-52E was purchased from the American Type Culture Collection (Manassas, VA, USA). NRK-52E cells were cultured in DMEM (Invitrogen, Carlsbad, CA, USA) with 10% fetal bovine serum (FBS, Invitrogen) at 37°C containing 5% CO_2_. NRK-52E cells after H/R treatment (H/R-treated NRK-52E cells) acted as the RI/RI model at the cellular level. Briefly, NRK-52E cells were exposed to hypoxia (94% N_2_, 5% CO_2_, and 1% O_2_) for 24 h followed by 12 h of reoxygenation (74% N_2_, 5% CO_2_, and 21% O_2_). The NRK-52E cells in the control culture served as the control group. Then, the H/R-treated NRK-52E cells were divided into three groups: H/R group (H/R-treated NRK-52E cells without treatment), NC group (H/R-treated NRK-52E cells which were treated with 0.9% sterile NaCl for 12 h before H/R treatment), and TGP group (H/R-treated NRK-52E cells which were treated with 10 *μ*g/mL TGP for 12 h before H/R treatment).

### 2.7. Cell Transfection

The pcDNA3.1 XIST (pcDNA-XIST), pcDNA-NC, miR-124-3p mimics, miR-NC, short hairpin- (sh-) ITGB1, and sh-NC were synthesized by GenePharma (Shanghai, China). H/R-treated NRK-52E cells grown to 85% confluence were transfected or cotransfected with these above agents using Lipofectamine 3000 (Invitrogen). The H/R-treated NRK-52E cells in the blank group did not receive any transfection.

### 2.8. Quantitative Real-Time Polymerase Chain Reaction (qRT-PCR)

The expression of XIST, miR-124-3p, and ITGB1 was measured by qRT-PCR as previously described [30]. Total RNA was extracted from tissues and cells using the TRIzol reagent (Invitrogen). Then, cDNA samples were attained through reverse transcription using a PrimeScript RT Reagent Kit (TaKaRa, Japan). Next, qRT-PCR was conducted on a 7500 Real-Time PCR System (Applied Biosystems, Waltham, MA, USA). Relative expression was calculated by the 2^-*ΔΔ*Ct^ method. GAPDH, U6, and *β*-actin were used for the normalization of XIST, miR-124-3p, and ITGB1, respectively. The primer sequences are shown in Table 1.

### 2.9. Quantitative Analysis of Proinflammatory Cytokines

NRK-52E cells were centrifuged at 5,000 × g at 4°C for 10 min, and the resulting supernatant was collected. The concentrations of interleukin- (IL-) 1*β*, IL-6, and tumor necrosis factor-*α* (TNF-*α*) in the serum and cell supernatant were measured using enzyme-linked immunosorbent assay (ELISA) kits (Sigma, St. Louis, MO, USA). The absorbance of each assay well was measured at 450 nm by a microplate reader (Molecular Devices, Sunnyvale, CA, USA).

### 2.10. MTT Assay

NRK-52E cells were seeded into 96-well plates (2 × 10^3^ cells/well) and cultured with 5% CO_2_ at 37°C for 72 h. Cell viability was measured using the MTT cell proliferation assay kit (Sigma) according to the guidelines.

### 2.11. Flow Cytometer

NRK-52E cells were trypsinized and washed with phosphate-buffered saline twice. Then, NRK-52E cells were stained by using Annexin V-FITC and propidium iodide (Invitrogen) for 15 min in a dark room. Afterwards, the apoptotic cells were observed with a MUSE™ flow cytometer (Beckman, Miami, FL, USA).

### 2.12. Western Blot

Total proteins were extracted from NRK-52E cells and then transferred into SDS-PAGE. The separated protein was transferred onto polyvinylidene fluoride membranes, blocked with 5% skimmed milk, and incubated overnight at 4°C with primary anti-ITGB1 antibody (0.04-0.4 *μ*g/mL, HPA059297MSDS, Sigma) or *β*-actin (1 : 4000, SAB2701711MSDS, Sigma). Afterwards, the membranes were subjected to HRP-labeled goat anti-rabbit IgG (1 : 5000, 12-348MSDS, Sigma) secondary antibody at 25°C for 1 h. The immunoblots were measured by ECL system and quantified by Image Lab software (Bio-Rad, Hercules, CA, USA).

### 2.13. Dual-Luciferase Reporter Assay

The potential binding sites of XIST and miR-124-3p or ITGB1 and miR-124-3p were predicted by starBase or TargetScan, respectively. XIST and ITGB1 with WT or MUT miR-124-3p-binding sites were generated and fused to the psiCHECK-2 vectors (YouBio, Hunan, China). NRK-52E cells were cotransfected with the above luciferase vectors and miR-NC or miR-124-3p mimics using Lipofectamine 3000 (Invitrogen).

### 2.14. Statistical Analysis

Data statistical analysis was performed using GraphPad Prism 7.0 (GraphPad, San Diego, CA, USA). Data were presented as mean ± standard deviation. The differences between two groups or among multiple groups were assessed by Student's *t*-test or one-way ANOVA followed by Tukey's post hoc test. Differences were considered statistically significant at *P* < 0.05.

## 3. Results

### 3.1. TGP Alleviated Renal Dysfunction and Inflammation in RI/RI

**Figure 1:**
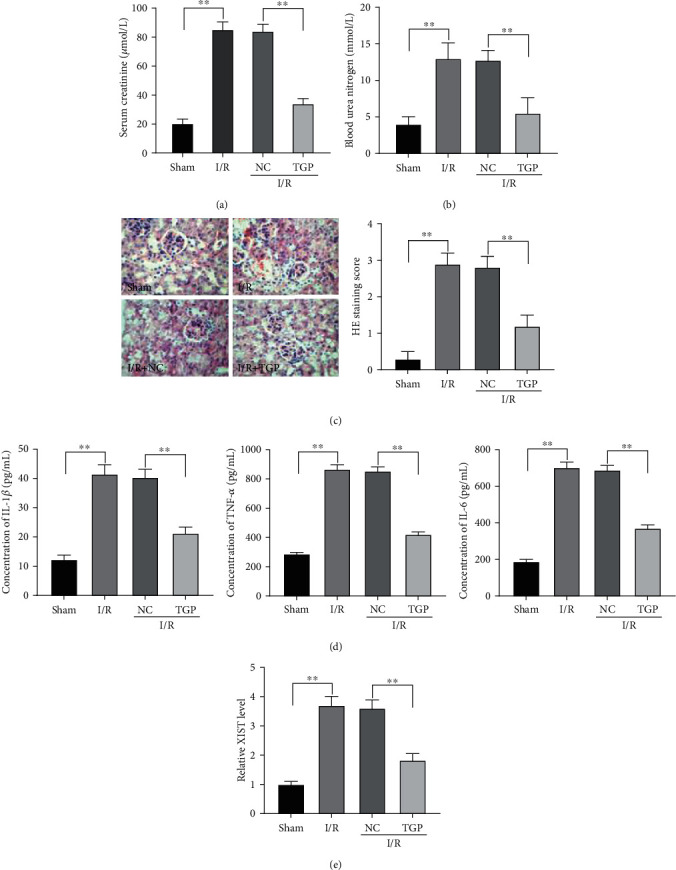
Total glucosides of paeony (TGP) alleviated renal dysfunction and inflammation in renal ischemia/reperfusion injury (RI/RI): (a) serum creatinine (SCr) level was significantly higher in the I/R group than in the sham group and was reduced by TGP treatment, ^∗∗^*P* < 0.01 vs. sham and NC; (b) serum blood urea nitrogen (BUN) level was notably increased in rat RI/RI model and was markedly decreased by TGP treatment, ^∗∗^*P* < 0.01 vs. sham and NC; (c) the histological injury of renal tissues was detected by haematoxylin-eosin (HE) staining, original magnifications: 400x, ^∗∗^*P* < 0.01 vs. sham and NC; (d) the concentrations of IL-1*β*, TNF-*α*, and IL-6 in renal tissues were determined by enzyme-linked immunosorbent assay (ELISA), ^∗∗^*P* < 0.01 vs. sham and NC; (e) the expression of XIST in renal tissues was examined by qRT-PCR, ^∗∗^*P* < 0.01 vs. sham and NC.

We established a RI/RI model in rats. As shown in Figures 1(a) and 1(b), the levels of SCr and BUN were considerably upregulated in the I/R group compared to the sham group (*P* < 0.01). Pretreatment of TGP dramatically decreased the elevation of SCr and BUN levels after RI/RI (*P* < 0.01). A 0- to 4-point scoring system (HE staining score) was used to assess histological injury. The results revealed that HE staining score was higher in the I/R group than in the sham group, and TGP could markedly reduce the HE staining score after RI/RI (*P* < 0.01, Figure 1(c)). In addition, the concentrations of proinflammatory cytokines IL-1*β*, TNF-*α*, and IL-6 obviously increased after RI/RI, whereas treatment with TGP strikingly declined these concentrations (*P* < 0.01, Figure 1(d)). Interestingly, XIST expression was notably enhanced in the rat RI/RI model and was markedly inhibited by TGP treatment (*P* < 0.01, Figure 1(e)).

### 3.2. TGP Increased Viability and Inhibited Apoptosis and Inflammation of Renal Cells after H/R Treatment

**Figure 2:**
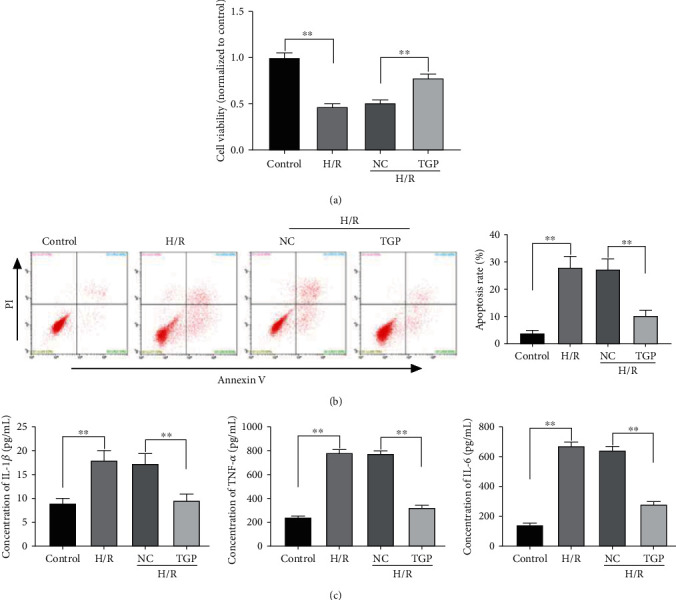
Total glucosides of paeony (TGP) increased viability and inhibited apoptosis and inflammation of renal cells after hypoxia/reoxygenation (H/R) treatment: (a) the viability of NRK-52E cells was measured by MTT assay, ^∗∗^*P* < 0.01 vs. control and NC; (b) the apoptosis rate of NRK-52E cells was determined by flow cytometry, ^∗∗^*P* < 0.01 vs. control and NC; (c) ELISA assay was performed to confirm the concentrations of IL-1*β*, TNF-*α*, and IL-6 in NRK-52E cells, ^∗∗^*P* < 0.01 vs. control and NC.

To construct the RI/RI model at the cellular level, NRK-52E cells were subjected to 24 h of hypoxia followed by 12 h of reoxygenation. The MTT assay discovered that the viability of NRK-52E cells was visibly reduced after H/R treatment, while TGP could significantly increase viability of NRK-52E cells after H/R treatment (*P* < 0.01, Figure 2(a)). In contrast, in cultured NRK-52E cells, H/R treatment obviously elevated the apoptosis rate, and the cell apoptosis rate in the TGP group was lower than that in the NC group (*P* < 0.01, Figure 2(b)). Furthermore, the concentrations of IL-1*β*, TNF-*α*, and IL-6 obviously increased in NRK-52E cells after H/R treatment, and TGP could clearly decline the elevated concentration of proinflammatory cytokines caused by H/R treatment (*P* < 0.01, Figure 2(c)).

### 3.3. TGP Increased Viability and Attenuated Apoptosis and Inflammation of H/R-Treated Renal Cell via Inhibiting XIST

**Figure 3:**
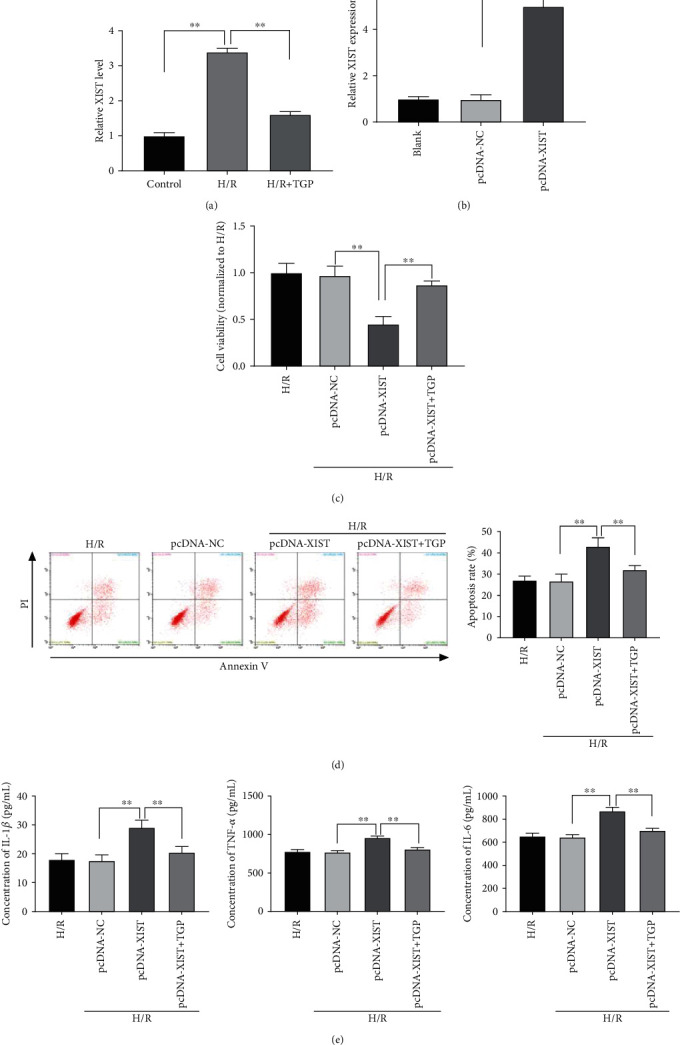
Total glucosides of paeony (TGP) increased viability and attenuated apoptosis and inflammation of hypoxia/reoxygenation- (H/R-) treated renal cell via inhibiting XIST: (a) qRT-PCR was performed to assess the expression of XIST in the control, H/R, and H/R+TGP group, ^∗∗^*P* < 0.01 vs. control and H/R; (b) qRT-PCR was used to evaluate the transfection efficiency of pcDNA-NC and pcDNA-XIST in NRK-52E cells, ^∗∗^*P* < 0.01 vs. pcDNA-NC; (c) the viability of NRK-52E cells after H/R treatment was detected by MTT assay, ^∗∗^*P* < 0.01 vs. pcDNA-NC and pcDNA-XIST; (d) flow cytometry assay was used to determine the apoptosis rate of NRK-52E cells after H/R treatment, ^∗∗^*P* < 0.01 vs. pcDNA-NC and pcDNA-XIST; (e) the concentrations of IL-1*β*, TNF-*α*, and IL-6 in NRK-52E cells after H/R treatment were examined by ELISA, ^∗∗^*P* < 0.01 vs. pcDNA-NC and pcDNA-XIST.

To confirm the expression of XIST under RI/RI *in vitro*, qRT-PCR was implemented. Results discovered that XIST expression was highly expressed in NRK-52E cells after H/R treatment, whereas treatment with TGP obviously downregulated the XIST expression (*P* < 0.01, Figure 3(a)). To further determine the biological function of XIST in RI/RI *in vitro*, XIST was enhanced by the transfection of pcDNA-XIST (*P* < 0.01, Figure 3(b)). As illustrated in Figures 3(c)–3(e), XIST upregulation strikingly declined the viability, as well as visibly elevated the apoptosis rate and proinflammatory cytokines (IL-1*β*, TNF-*α*, and IL-6) in NRK-52E cells after H/R treatment (*P* < 0.01). Pretreatment of TGP could not only notably mitigate the inhibitory effect of pcDNA-XIST on the viability of H/R-treated NRK-52E cells but also markedly weaken the promoting effect of pcDNA-XIST on the apoptosis and inflammation in H/R-treated NRK-52E cells (*P* < 0.01).

### 3.4. XIST Reversely Modulated miR-124-3p Expression

**Figure 4:**
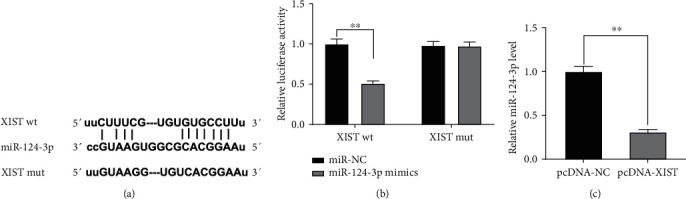
XIST reversely modulated miR-124-3p expression: (a) starBase exhibited the predicted binding site between XIST and miR-124-3p; (b) relative luciferase activity in NRK-52E cells was measured by dual-luciferase reporter assay, ^∗∗^*P* < 0.01 vs. miR-NC; (c) the expression of miR-124-3p in NRK-52E cells was detected by qRT-PCR, ^∗∗^*P* < 0.01 vs. pcDNA-NC.

To investigate the mechanism of the function of XIST on RI/RI *in vitro*, we sifted miRNAs that have correlative base pairing with XIST using starBase, and miR-124-3p was predicted as a potential target of XIST (Figure 4(a)). Then, dual-luciferase reporter assay confirmed that miR-124-3p overexpression considerably inhibited the relative luciferase activity of the reporter with XIST WT in NRK-52E cells (*P* < 0.01, Figure 4(b)). Moreover, pcDNA-XIST could visibly downregulate miR-124-3p expression in NRK-52E cells (*P* < 0.01, Figure 4(c)).

### 3.5. ITGB1 Served as a Target of miR-124-3p

**Figure 5:**
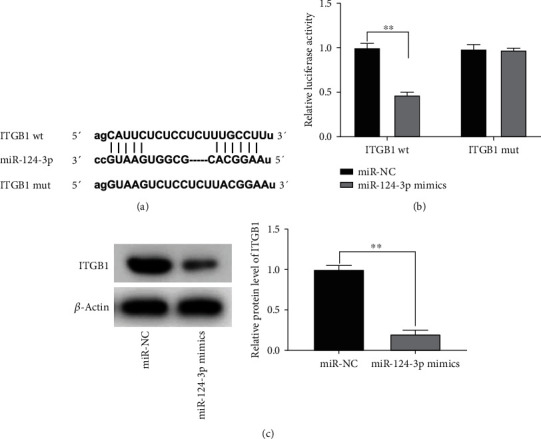
ITGB1 served as a target of miR-124-3p: (a) TargetScan showed the predicted binding site between ITGB1 and miR-124-3p; (b) dual-luciferase reporter assay was performed to measure the relative luciferase activity in NRK-52E cells, ^∗∗^*P* < 0.01 vs. miR-NC; (c) the protein expression of ITGB1 in NRK-52E cells was measured by western blot, ^∗∗^*P* < 0.01 vs. miR-NC.

Bioinformatics analysis by using TargetScan revealed that miR-124-3p binds to 3′UTR of ITGB1 mRNA (Figure 5(a)). A dual luciferase reporter assay indicated that the luciferase activity of NRK-52E cells treated with ITGB1 3′UTR-WT reporter was decidedly attenuated by miR-124-3p mimics (*P* < 0.01, Figure 5(b)). Additionally, miR-124-3p could markedly reduce the protein expression of ITGB1 in NRK-52E cells (*P* < 0.01, Figure 5(c)).

### 3.6. XIST Decreased Viability and Promoted Apoptosis and Inflammation of Renal Cells after H/R Treatment via Regulating miR-124-3p/ITGB1 Axis

**Figure 6:**
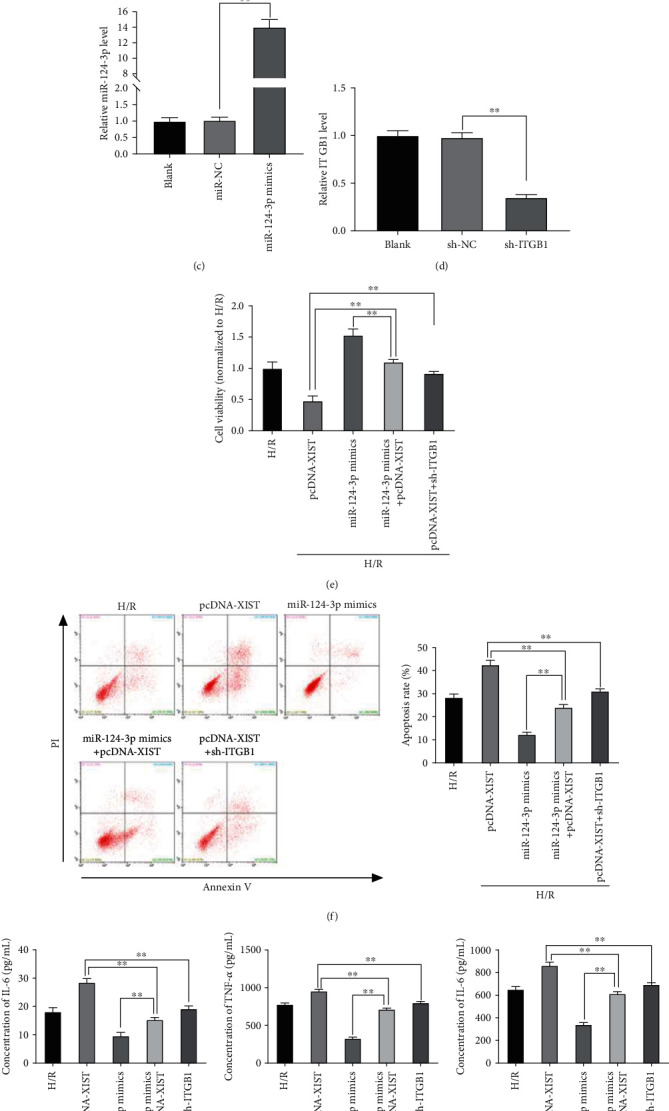
XIST decreased viability and promoted apoptosis and inflammation of renal cells after hypoxia/reoxygenation (H/R) treatment via regulating miR-124-3p/ITGB1 axis: (a) qRT-PCR was performed to measure the expression of miR-124-3p in the control, H/R, and H/R+TGP groups, ^∗∗^*P* < 0.01 vs. control and H/R; (b) ITGB1 expression in the control, H/R, and H/R+TGP groups was determined by qRT-PCR, ^∗∗^*P* < 0.01 vs. control and H/R; (c) the transfection efficiencies of miR-NC and miR-124-3p mimics were demonstrated by using qRT-PCR, ^∗∗^*P* < 0.01 vs. miR-NC; (d) qRT-PCR was performed to evaluate the transfection efficiency of sh-NC and sh-ITGB1, ^∗∗^*P* < 0.01 vs. sh-NC; (e) overexpression of miR-124-3p or inhibition of ITGB1 reversed the inhibitory effect of XIST overexpression on viability of NRK-52E cells after H/R treatment, ^∗∗^*P* < 0.01 vs. pcDNA-XIST and miR-124-3p mimics; (f, g) the promoting effect of XIST overexpression on apoptosis and inflammation of H/R-treated NRK-52E cells was mitigated by miR-124-3p overexpression or ITGB1 knockdown, ^∗∗^*P* < 0.01 vs. pcDNA-XIST and miR-124-3p mimics.

As exhibited in Figure 6(a), the miR-124-3p expression was considerably decreased in H/R-treated NRK-52E cells, and TGP could obviously rescue the miR-124-3p downregulation after H/R treatment (*P* < 0.01). Besides, ITGB1 expression was markedly upregulated in the H/R group compared with the control group, while TGP dramatically reversed the ITGB1 overexpression after H/R treatment (*P* < 0.01, Figure 6(b)). Then, the miR-124-3p expression was obviously enhanced by the transfection of miR-124-3p mimics (*P* < 0.01, Figure 6(c)), and the ITGB1 expression was obviously inhibited by the transfection of sh-ITGB1 (*P* < 0.01, Figure 6(d)). To further investigate the molecular mechanism by which XIST overexpression promoted the RI/RI *in vitro*, rescue experiments were performed. As shown in Figures 6(e)–6(g), the miR-124-3p overexpression or ITGB1 inhibition could not only notably rescue the inhibitory effect of pcDNA-XIST on the viability of NRK-52E cells after H/R treatment but also markedly reverse the promoting effect of pcDNA-XIST on the apoptosis and inflammation of NRK-52E cells after H/R treatment (*P* < 0.01).

## 4. Discussion

RI/RI, and subsequent kidney damage, may be attributed to inflammation, apoptosis, and oxidative stress [31]. TGP has been demonstrated to play a key role in diverse renal diseases [32, 33]. In this study, the serum levels of SCr, BUN, proinflammatory cytokines, and histological injury were obviously decreased by TGP treatment in RI/RI rat model. The function of TGP was similar to previously described TCMs. For instance, lavender oil can exert protective effects against RI/RI through reducing proinflammatory cytokine levels and tissue damage [34]. Treatment with proanthocyanidin declines the levels of SCr and BUN and retards histological alterations in the RI/RI rat [35]. Catalpol protects mice against RI/RI through downregulating SCr and BUN levels and suppressing inflammation [36]. Above all, we suggest that TGP has an ameliorating effect on RI/RI *in vivo*. To further verify the role of TGP, RI/RI was simulated by H/R treatment in NRK-52E cells. Our results displayed that TGP increased viability and inhibited apoptosis and inflammation of renal cells after H/R treatment. Similarly, multiple TCMs have been used as the antiapoptosis and anti-inflammation drug for the treatment of RI/RI. Baicalin alleviates RI/RI via attenuating mitochondria-mediated apoptosis and inflammatory response of renal cells [37]. Resveratrol represses inflammatory responses and apoptosis and elevates viability of renal cells in RI/RI [38]. Taken together, we indicate that TGP has a protective effect against RI/RI both *in vivo* and *in vitro*.

Enhanced expression of lncRNAs, such as lncRNA PRINS [39], lncRNA Malat1 [40], and lncRNA TUG1 [41], has been discovered in RI/RI. Here, XIST expression was notably upregulated in the rat RI/RI model and H/R-treated renal cells. Reducing cell apoptosis and inhibiting the inflammatory responses have become a pivotal focus in treating IRI [42]. Interference of XIST exerts protective functions in IRI. XIST knockdown restrains cell apoptosis and autophagy in myocardial tissues of IRI mice [43]. XIST silencing reduces the levels of TNF-*α* and IL-6 in a rat acute kidney injury model caused by I/R [17]. In this study, the elevation of XIST in both the rat RI/RI model and H/R-treated renal cells was markedly downregulated by TGP treatment. In addition, XIST overexpression decreased viability and increased apoptosis and inflammation of renal cells after H/R treatment, and TGP treatment obviously reversed the effects of XIST overexpression exerted in H/R-treated renal cells. Taken together, we indicate that TGP may alleviate RI/RI *in vitro* through inhibiting XIST.

Numerous studies have reported that lncRNAs interact with miRNAs, participating in the regulation of RI/RI. For example, lncRNA GAS5 accelerates apoptosis via competitively sponging miR-21, thus contributing to RI/RI in renal cells [44]. lncRNA TUG1 silencing impedes I/R-induced apoptosis and inflammation by decreasing miR-449b-5p in RI/RI [41]. In this study, miR-124-3p was regarded as a target of XIST, and its expression was negatively related to XIST. Increasing evidences have exhibited that miR-124 is downregulated and plays a critical role in the regulation of RI/RI. Sinomenine facilitates cell proliferation and represses apoptosis of renal cells after H/R treatment via enhancing miR-124 expression [45]. miR-124 expression is declined in the RI/RI model, and miR-124 overexpression takes part in the prevention and treatment of RI/RI [46]. Here, we observed that miR-124-3p was downregulated in H/R-treated renal cells, and TGP could obviously rescue the miR-124-3p downregulation. Interestingly, miR-124-3p overexpression elevated cell viability and mitigated cell apoptosis and inflammation in H/R-treated renal cells. Moreover, pcDNA-XIST obviously reversed the effects of miR-124-3p overexpression exerted in H/R-treated renal cells. Thus, XIST may promote RI/RI *in vitro* via competitively binding to miR-124-3p.

miRNAs exert its function through targeting ITGB1 in several diseases [47, 48]. Notably, ITGB1 was inversely correlated with miR-124-3p in nasopharyngeal carcinoma [49]. In this study, ITGB1 was a target of miR-124-3p, and the expression of ITGB1 was inversely related to miR-124-3p. Considering the interaction of XIST/miR-124-3p, we hypothesize that XIST may mediate ITGB1 expression in RI/RI. It has been documented that ITGB1 inhibition can attenuate the development of kidney diseases. For instance, inhibition of ITGB1 hinders tPA-mediated fibroblast proliferation in chronic kidney diseases [50]. Inactivation of ITGB1 leads to preservation of normal renal function and suppression of fibrosis in polycystic kidney disease [51]. Rosiglitazone inhibits the high glucose-induced apoptosis of proximal tubular cell by suppressing ITGB1 expression [52]. Here, ITGB1 was upregulated in H/R-treated renal cells, and TGP could visibly reverse the ITGB1 overexpression. Knockdown of ITGB1 not only rescued the reduction effects on viability but also weakened the promoting effects on cell apoptosis and inflammation caused by XIST overexpression in H/R-treated renal cells. To sum up, XIST mitigated cell viability and elevated cell apoptosis and inflammation in H/R-treated renal cells through targeting the miR-124-3p/ITGB1 axis.

## 5. Conclusions

In conclusion, the present study confirmed that TGP protected against RI/RI through alleviating renal dysfunction and inflammation in rat. Furthermore, TGP restrained apoptosis and inflammation of H/R-treated renal cells by targeting the XIST/miR-124-3p/ITGB1 axis. TGP could be a promising therapeutic drug for RI/RI.

## Figures and Tables

**Table 1:** Primer sequences.

Name of primer	Sequences (5′-3′)
XIST-F	AGCTCCTCGGACAGCTGTAA
XIST-R	CTCCAGATAGCTGGCAACC
GAPDH-F	AATCCCATCACCATCTTCCAG
GAPDH-R	GAGCCCCAGCCTTCTCCAT
miR-124-3p-F	ACAGGCTAAGGCTCCCAGTGA A
miR-124-3p-R	CGCAGGGTCCGAGGTATTC
U6-F	CTCGCTTCGGCAGCACA
U6-R	AACGCTTCACGA ATTTGCGT
ITGB1-F	ATCCCAGAGGCTCCAAAGAT
ITGB1-R	CCC CTGATCTTAATCGCAAA
*β*-Actin-F	CATGTACGTTGCTATCCAGGC
*β*-Actin-R	CTCCTTAATGTCACGCACGAT

## Data Availability

All data generated or analyzed during this study are included in this published article.
